# Assessment of pharmacologically induced changes in canine kidney function by multiparametric magnetic resonance imaging and contrast enhanced ultrasound

**DOI:** 10.3389/fvets.2024.1406343

**Published:** 2024-06-20

**Authors:** Amber Hillaert, Luis Carlos Sanmiguel Serpa, Stephanie Bogaert, Bart J. G. Broeckx, Myriam Hesta, Eva Vandermeulen, Jolien Germonpré, Emmelie Stock, Pim Pullens, Katrien Vanderperren

**Affiliations:** ^1^Department of Morphology, Imaging, Orthopedics, Rehabilitation and Nutrition, Faculty of Veterinary Medicine, Ghent University, Merelbeke, Belgium; ^2^Department of Medical Imaging, Ghent University Hospital, Ghent, Belgium; ^3^Ghent Institute for Functional and Metabolic Imaging, Ghent University, Ghent, Belgium; ^4^Department of Diagnostic Sciences, Faculty of Medicine and Health Sciences, Ghent University, Ghent, Belgium; ^5^Department of Veterinary and Biosciences, Faculty of Veterinary Medicine, Ghent University, Merelbeke, Belgium; ^6^Institute of Biomedical Engineering and Technology, Faculty of Engineering and Architecture, Ghent University, Ghent, Belgium

**Keywords:** kidney, perfusion, oxygenation, ASL-MRI, BOLD-MRI, DCE-MRI, CEUS, dog

## Abstract

**Introduction:**

Dynamic contrast-enhanced (DCE) MRI and arterial spin labeling (ASL) MRI enable non-invasive measurement of renal blood flow (RBF), whereas blood oxygenation level-dependent (BOLD) MRI enables non-invasive measurement of the apparent relaxation rate (R2*), an indicator of oxygenation. This study was conducted to evaluate the potential role of these MRI modalities in assessing RBF and oxygenation in dogs. The correlation between contrast-enhanced ultrasound (CEUS) and the MRI modalities was examined and also the ability of the MRI modalities to detect pharmacologically induced changes.

**Methods:**

RBF, using CEUS, ASL- and DCE-MRI, as well as renal oxygenation, using BOLD-MRI of eight adult beagles were assessed at two time-points, 2–3 weeks apart. During each time point, the anesthetized dogs received either a control (0.9% sodium chloride) or a dopamine treatment. For each time point, measurements were carried out over 2 days. An MRI scan at 3 T was performed on day one, followed by CEUS on day two.

**Results:**

Using the model-free model with caudal placement of the arterial input function (AIF) region of interest (ROI) in the aorta, the DCE results showed a significant correlation with ASL measured RBF and detected significant changes in blood flow during dopamine infusion. Additionally, R2* negatively correlated with ASL measured RBF at the cortex and medulla, as well as with medullary wash-in rate (WiR) and peak intensity (PI). ASL measured RBF, in its turn, showed a positive correlation with cortical WiR, PI, area under the curve (AUC) and fall time (FT), and with medullary WiR and PI, but a negative correlation with medullary rise time (RT). During dopamine infusion, BOLD-MRI observed a significant decrease in R2* at the medulla and entire kidney, while ASL-MRI demonstrated a significant increase in RBF at the cortex, medulla and the entire kidney.

**Conclusion:**

ASL- and BOLD-MRI can measure pharmacologically induced changes in renal blood flow and renal oxygenation in dogs and might allow detection of changes that cannot be observed with CEUS. However, further research is needed to confirm the potential of ASL- and BOLD-MRI in dogs and to clarify which analysis method is most suitable for DCE-MRI in dogs.

## 1 Introduction

Acute kidney injury (AKI) and chronic kidney disease (CKD) are common conditions that compromise renal function and affect both humans and dogs (1–4). As the glomerular filtration rate (GFR) is directly proportional to functional renal mass, it is regarded as a highly sensitive and specific indicator of renal function impairment (5). The GFR can be assessed in a number of ways using both direct and indirect measurement techniques. Direct measurement of GFR based on the clearance of a filtration marker is considered the gold standard, but this method is cumbersome and can only assess global renal function (5). Instead, GFR is often measured indirectly using surrogate markers (e.g., blood urea nitrogen and creatinine) in routine veterinary practice because of their ease of use (5). A major drawback of these commonly employed surrogate markers, however, is their inability to identify kidney injury at an early stage (5). The imaging technique scintigraphy, on the other hand, makes it possible to determine, in addition to the total GFR, the individual contribution of both kidneys within a short time frame of 20 to 30 min (6, 7). Renal scintigraphy in dogs relies on the detection of gamma radiation from the injected radiopharmaceutical technetium-99 m-diethylenetriaminepetaacetic acid (^99m^Tc-DTPA), which the kidney removes from the blood via glomerular filtration (6, 7). The GFR is calculated using a regression equation relating the percentage dose uptake of ^99m^Tc-DTPA by each kidney to the GFR measured with plasma inulin clearance (6, 7). In dogs, scintigraphy has been shown to be equivalent to inulin clearance in determining GFR (8, 9). Nevertheless, scintigraphy has some disadvantages as well, such as the need for radioactive isotopes, the need for short-term patient isolation, and the fact that it is only available in veterinary specialist clinics with advanced technological capabilities (7).

In addition to GFR, renal perfusion and oxygenation are essential factors to consider in the renal function assessment (10, 11). The development and progression of various forms of AKI and CKD are significantly influenced by perfusion impairment and hypoxia of the renal tissue (10, 11). In the early stages of renal disease, hypoperfusion and hypoxia may even be present without other signs of renal dysfunction (12, 13). Assessment of tissue perfusion and oxygenation is complicated by the lack of gold standard techniques and limitations of current reference techniques (14, 15). Furthermore, this complicates the evaluation of potential new techniques to assess tissue perfusion and oxygenation (14, 15).

Contrast-enhanced ultrasound (CEUS) is an imaging technique that enables assessment of microvascular perfusion by intravenous administration of an ultrasound contrast agent (16). Ultrasound contrast agents consist of tiny, encapsulated gas bubbles that are contained within the intravascular space and reflect ultrasound waves as harmonic signals (16). Several studies have demonstrated the potential of CEUS in evaluating kidney diseases in humans and animals. Among others, CEUS was found to be a sensitive method to assess the changes in renal microvascular perfusion in canine acute kidney disease (17), as well as in chronic kidney disease in cats (18) and humans (19). A drawback of CEUS, however, is its high intrinsic variability (20).

Functional magnetic resonance imaging (fMRI), on the other hand, is an emerging imaging technique that can non-invasively assess both renal structure and function of the entire renal parenchyma. Using fMRI modalities such as dynamic contrast-enhanced (DCE) MRI, arterial spin labeling (ASL) and blood oxygenation level-dependent (BOLD) MRI, multiple functional kidney parameters can be measured in a single scan session, including perfusion, glomerular filtration and oxygenation (21).

With DCE MRI, a gadolinium contrast agent is injected intravenously and the contrast uptake is analyzed based on a pharmacokinetic (PK) model to determine RBF and GFR of each kidney (21, 22). Although there have been some concerns regarding the safety of gadolinium contrast, especially in patients with renal impairment, low doses used in clinical settings have been demonstrated to be not harmful (23). Among the advantages of DCE-MRI are its lack of radioactive radiation, its limited duration, the ability to determine single kidney GFR, and the possibility to use other MRI modalities simultaneously (23). DCE-MRI has been successfully used to measure renal function parameters (GFR and RBF) in humans (24) and animals, such as pigs (25), rabbits (26, 27), rats (28, 29), and mice (30, 31). So far, DCE-MRI has only been used in a limited number of studies on dogs. Aumann et al. (32) evaluated the feasibility of renal perfusion assessment with DCE-MRI in dogs by comparison with renal artery blood flow measurements using an implanted flow probe. Another study demonstrated that quantitative renal perfusion measurements of DCE-MRI can be used for the evaluation of renal artery stenosis in dogs (33). One canine study has described the successful quantification of the single kidney GFR using DCE-MRI in dogs (34).

ASL-MRI enables tissue perfusion quantification by using magnetically labeled blood water protons as an endogenous contrast agent (15). A labeled image is first collected in which a radiofrequency pulse alters the longitudinal magnetization of arterial blood flowing into the kidney. An image with an unchanged magnetization is obtained next as a control. By subtracting the label from the control image, a perfusion-weighted image is created where signal intensity is proportional to perfusion (15). The perfusion weighted images are analyzed by a kinetic model to quantify renal perfusion and get a quantitative perfusion map (15). The feasibility of RBF determination by ASL-MRI of kidneys with both normal and altered function was demonstrated by previous studies in humans (35, 36), pigs (37, 38), rabbits (27, 39) and rats (40, 41). There was a good correlation between ASL-MRI measurements of renal blood flow and measurements from alternative methods to evaluate renal blood flow. For example, ASL-MRI showed a good correlation with DCE-MRI (42), plasma clearance of *para*-aminohippuric acid (35) and technetium-99 m-mercaptoacetyltriglycine (^99m^Tc-MAG3) scintigraphy (43) in humans, with DCE-MRI in rabbits (27), and with microspheres and ultrasound flowmeter in pigs (37, 38). To our knowledge, no other researchers have conducted any studies on dogs with renal ASL-MRI.

In BOLD-MRI the paramagnetic effect of deoxyhemoglobin is used as an endogenous contrast agent (44). Tissue deoxyhemoglobin concentration rises as tissue oxygen content decreases, causing the effective transverse relaxation time (T2*) of adjacent water protons to decrease (44). The apparent relaxation rate R2* (1/T2*), the reciprocal of T2*, rises with increasing concentrations of deoxygenated hemoglobin and is a measure of tissue oxygenation (44). The sensitivity of BOLD-MRI to assess intrarenal oxygenation under physiological and various pathophysiological conditions has been demonstrated by several studies in humans (45–47) and animal models (pig (48, 49), rabbit (39, 50), rat (41, 51) and mice (52)). However, some studies have produced contradictory findings, showing no distinction between healthy and diseased kidneys (53, 54). In dogs, there has only been one prior study that used BOLD-MRI to quantify renal oxygenation to our knowledge (55). Lee et al. (55) studied the feasibility and repeatability of BOLD-MRI derived T2* between two sessions in eight healthy beagles under anesthesia before and after furosemide administration.

A comprehensive assessment of renal function can be obtained using a combination of several fMRI techniques in addition to morphological images, i.e., multiparametric MRI (56, 57). Multiparametric MRI has the potential to elucidate (patho) physiological processes of the kidney and evaluate treatment effects. A great deal remains to be discovered about the normal physiology of renal oxygenation and perfusion, as well as the causes and consequences of their dysregulation in renal disease (56, 57). Due to the invasive nature of the present experimental and transitional research methods, client-owned dogs and human volunteers cannot be enrolled in certain experiments (48). The main aim of this study is to evaluate fMRI’s potential value for the assessment of renal perfusion and oxygenation in dogs. The objective of this study was to investigate which DCE analysis method, based on RBF measurements using ASL-MRI, is most appropriate for evaluating RBF in dogs. This study also examined the correlation between measurements of CEUS and the fMRI modalities (ASL-, BOLD- and DCE-MRI). Furthermore, the ability of fMRI modalities to detect pharmacologically induced changes in renal parameters, perfusion and oxygenation, was evaluated.

## 2 Materials and methods

### 2.1 Dogs

This study used eight purpose-bred beagles, four males (two intact, two neutered) and four females (three intact, one spayed). Their age ranged from 4 to 9.6 years [mean ± standard deviation (SD), 5.4 ± 1.6 years] and their body weight ranged from 9.4 to 15.6 kg (mean ± SD, 12.5 ± 1.9 kg). A standard dry maintenance diet (Medium mature diet, Royal Canin^®^) was provided to the dogs as well as *ad libitum* access to tap water. A physical examination, thoracic radiography, abdominal ultrasonography, Doppler blood pressure measurement, and laboratory analyses including complete blood count, serum biochemistry profile and urinalysis (urine sediment, dipstick test, specific gravity (USG), urine protein:creatinine (UPC) ratio and bacterial culture) were used to establish the baseline health status. The study protocol was approved by the Animal Ethics Committee from the Faculty of Veterinary Medicine and the Faculty of Bioscience Engineering of Ghent University, Belgium (Approval number: EC2021-085).

### 2.2 Protocol

**Figure 1:**
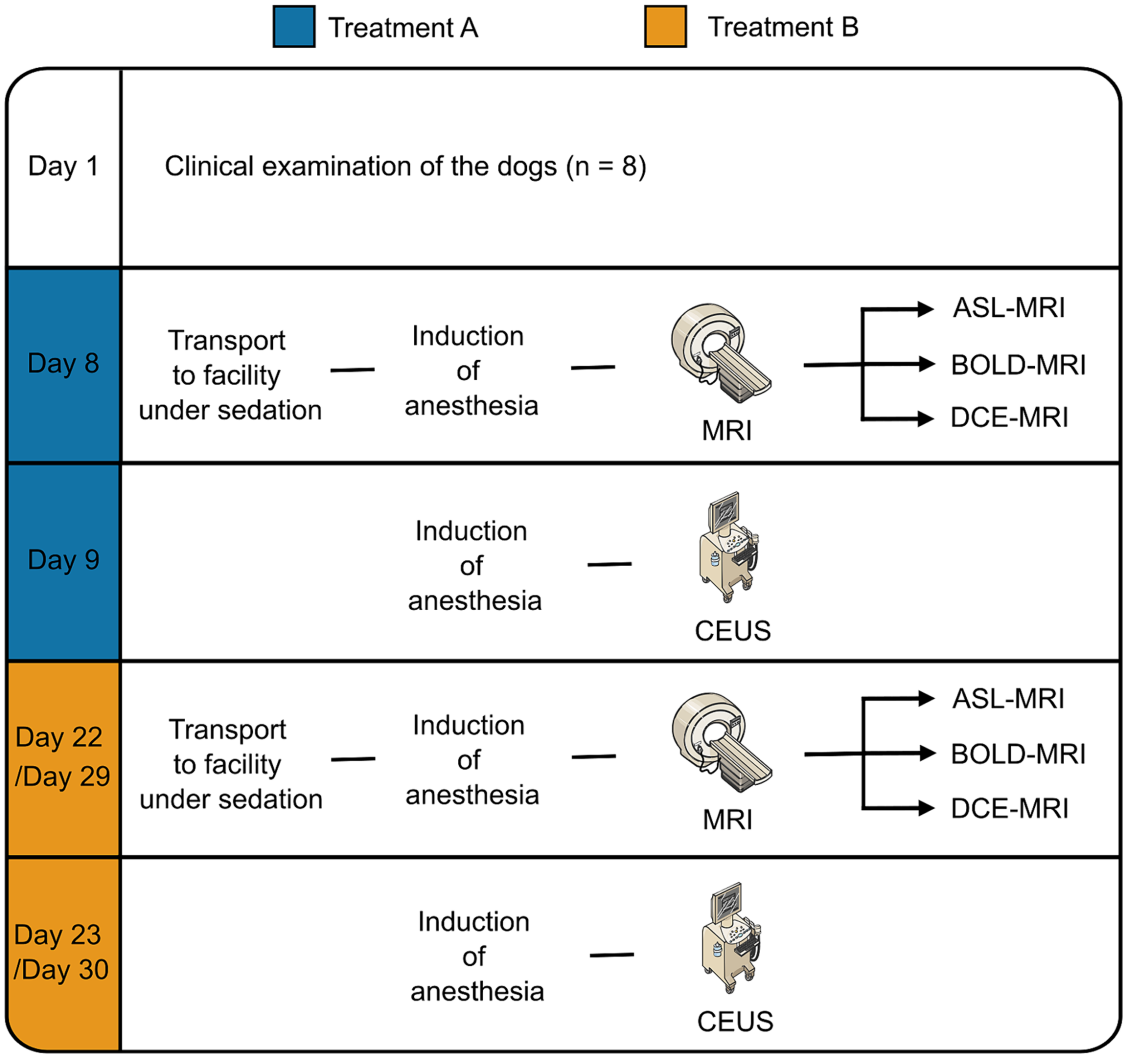
Flow chart of the study. Parts of the figure were drawn using modified pictures from Server Medical Art. Servier Medical Art by Servier is licensed under Creative Commons Attribution 4.0 International license (https://creativecommons.org/licenses/by/4.0/).

The renal perfusion and renal oxygenation of the dogs were assessed at two time-points, 2–3 weeks apart, using MRI and CEUS. During each time point, the dogs received either a control treatment with 0.9% sodium chloride (NaCl) or a treatment with dopamine. A different treatment was given to each dog at each time point, and the order of the treatments was randomized. For each time point, measurements were carried out over 2 days. An fMRI scan including ASL-, BOLD- and DCE-MRI was performed on day one, followed by CEUS on day two. The study protocol is illustrated in Figure 1.

### 2.3 Anesthesia

Food and water were withheld for 12 and 2 h before anesthesia induction. In the cephalic vein, a 22-gauge IV catheter was placed and butorphanol (0.2 mg/kg) (Dolorex^®^; MSD Animal Health, The Netherlands) was injected intravenously to induce sedation. Propofol (4–6 mg/kg IV) (PropoVet^®^; Zoetis, BE) was administered in conjunction with midazolam (0.2 mg/kg IV) (Midazolam Accord Healthcare^®^; Accord Healthcare Limited, UK) to induce anesthesia. Anesthesia was maintained with 1.2–1.4% isoflurane (Isoflutek^®^; Laboratorios Karizoo, Spain) in 100% oxygen, delivered via a circle rebreathing system, after dogs were intubated. Anesthesia monitoring included measurement of inspiratory and end-expiratory isoflurane and CO_2_ levels by capnography, invasive and/or non-invasive blood pressure measurement, and blood oxygen saturation and heart rate measurement by pulse oximetry.

Dogs received dopamine (3 μg/kg/min) (Dopaminehydrochloride Hikma^®^ 40 mg/mL; Hikma Pharmaceuticals, UK) or sodium chloride (0.9%) via an infusion pump at a rate of 3 mL/kg/h. The infusion started 5 min after the start of inhalation anesthesia and the evaluation of renal perfusion with the imaging techniques started 15 min after the start of the intravenous infusion of dopamine or 0.9% sodium chloride.

### 2.4 Contrast enhanced ultrasound

All scans were performed by a board-certified radiologist (E.S.) and an ECVDI-trained radiologist (E.V.), with 10 and 6 years of experience with CEUS, respectively. An ultrasound machine (iU22, Philips, Bothell, WA, United States) equipped with contrast specific software and a 12–5 MHz linear transducer was used for the CEUS examinations. The protocol used for the CEUS examinations was based on previous studies and was standardized (58, 59). The mechanical index was set to 0.08, in order to minimize microbubble destruction. Other standardized machine settings included focus (underneath the kidney), persistency (disabled) and dynamic range (50). The depth was adjusted to have the kidney in the field-of-view and the gain was optimized during the study, starting with a nearly anechoic image in contrast mode. The dogs were positioned in dorsal recumbency and coupling gel was applied to the area of shaved skin. The kidney’s longitudinal plane was imaged in dual screen-mode (side by side display of B- and contrast-mode images) and the transducer position was manually maintained throughout the examination. The 22-G catheter in the cephalic vein was fitted with a three-way stopcock. Using the three-way stopcock, sulfur hexafluoride-filled microbubbles (SonoVue^®^, Bracco Diagnostics Inc., Milan, Italy) were injected intravenously as a bolus (0.04 mL/kg) followed by 2 mL saline solution (NaCl 0.9%). Concurrent with injection of the contrast agent, a 90-s recording started. First, the left kidney was scanned, then the right kidney. Two injections were performed for the left kidney. Since the first injection typically results in lower enhancement (60), only images from the second injection were used for analysis. For the right kidney, one injection was administered. If technical difficulties were encountered during recording and images were therefore unsuitable for analysis, additional injections were performed. In all dogs, the injections were administered by the same person (A.H.) in a consistent way. Between two injections, the residual circulating microbubbles were eliminated by using high mechanical index pulses while scanning the caudal abdominal aorta and spleen. This was performed until a similar level of background echogenicity was obtained as before injection.

**Figure 2:**
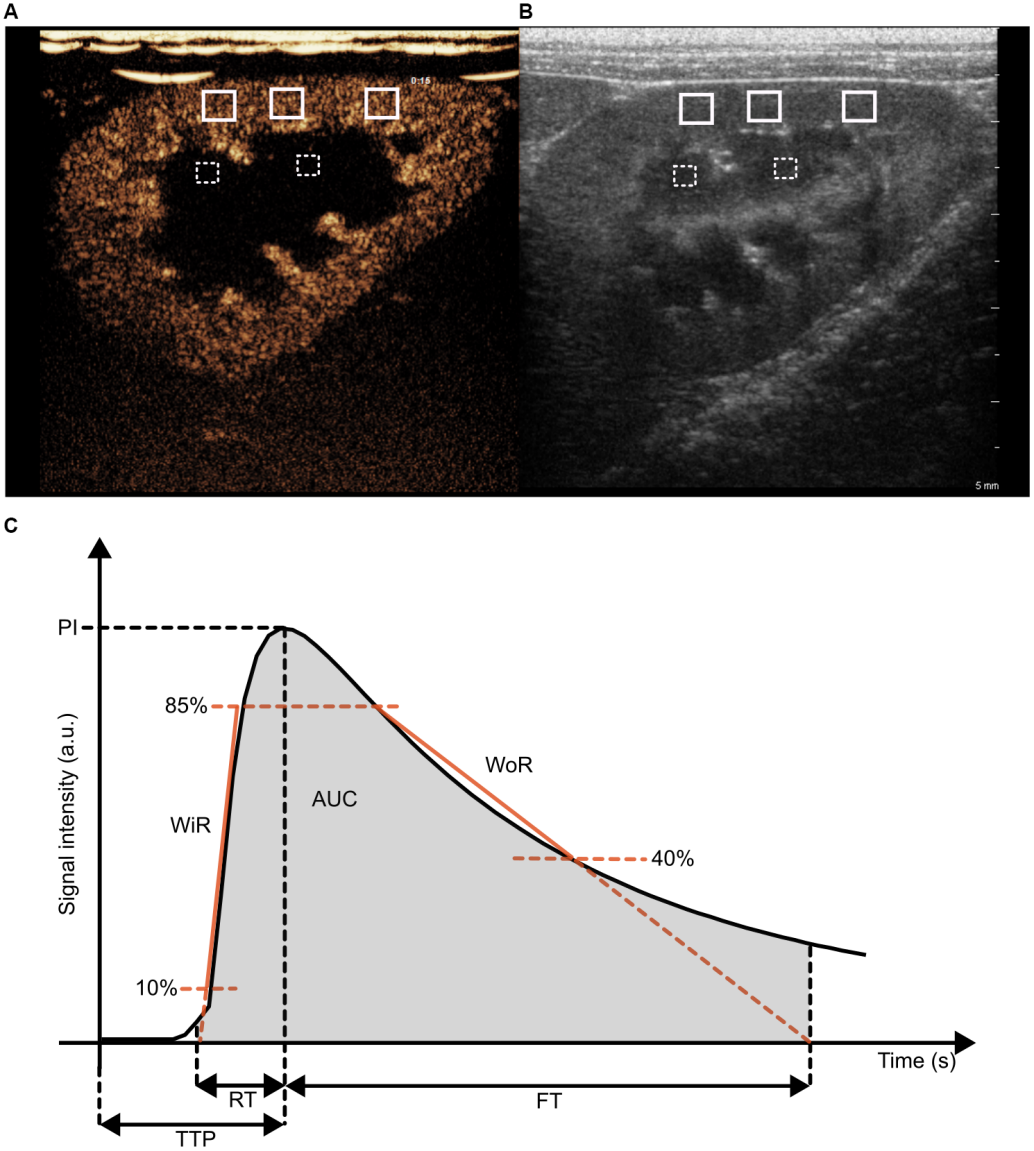
Illustration of regions-of-interest (ROIs) placement in the kidney on a contrast-enhanced ultrasound image **(A)** and a B-mode ultrasound image **(B)**, as well as of blood flow parameters in a time intensity curve **(C)**. **(A,B)** Three ROIs were drawn in the cortex (white, solid line) and two ROIs were drawn in the medulla (white, striped line). **(C)** Peak intensity (PI), total area under the curve (AUC), wash in rate (WiR), wash-out rate (WoR), time to peak (TTP), rise time (RT) and fall time (FT).

Quantitative analysis of the recordings was performed with dedicated software (QLAB, Philips Healthcare). Six regions of interest (ROIs) were manually positioned: 3 in the cortex, 2 in the medulla and 1 surrounding the entire kidney. Cortical and medullary ROIs were rectangular and had a size of 10 (± 0.20) mm^2^ and 6 (± 0.20) mm^2^, respectively (Figures 2A,B). Placement of the cortical and medullary ROIs was done at the same organ depth in a region with homogeneous enhancement, avoiding the inclusion of arteries. A time-intensity curve (TIC) was generated automatically for each ROI by the software. An average was calculated for the 3 ROIs in the cortex and 2 ROIs in the medulla. From the raw data, the following perfusion variables were calculated: peak intensity (PI), wash-in rate (WiR), wash-out rate (WoR), rise time (RT), fall time (FT), time to peak (TTP), total area under the curve (AUC) (Figure 2C). Blood volume is reflected by intensity-related parameters like PI, AUC, and WiR, whereas blood velocity is reflected by time-related parameters like TPP, RT, and FT (16).

The PI was defined as the maximum signal intensity minus the pre-rise baseline intensity. The WiR refers to the slope of the linear regression line for the data from 10% above baseline intensity to 85% of peak intensity. WoR refers to the slope of the linear regression line for data after the peak intensity, i.e., from 85 to 40% of peak intensity. TI is the time point at which the ascending linear regression line (10–85%) intersects the x-axis. TO is the time point at which the descending linear regression line (85–40%) intersects the x-axis. RT was defined as the time from the onset of enhancement to peak enhancement. FT was defined as the time from peak enhancement to TO. TTP was defined as the time between contrast injection and peak enhancement. AUC refers to the area under the time-intensity curve from timepoints TI to TO.

### 2.5 Magnetic resonance imaging scan protocol

All MRI examinations were performed with a 3 T MRI scanner (Siemens 3 T Magnetom Prisma Fit, Siemens AG, Healthcare Sector, Erlangen, Germany) running VE11C, using an 18 Channel Body Coil (Body 18, Siemens AG, Healthcare Sector, Erlangen, Germany). The dogs were positioned in dorsal recumbency with their head facing the MRI control room. A localizer sequence was acquired to determine the kidneys’ location and support scan planning.

#### 2.5.1 Arterial spin labeling

ASL images were acquired by using a Flow-sensitive Alternating Inversion Recovery (FAIR) labeling scheme with Q2TIPS (Siemens Work In Progress sequence). The readout parameters of the FAIR Q2TIPS ASL sequence were: imaging matrix 64 × 64, field of view (FOV) 272 × 136 mm^2^, voxel size 2.1 × 2.1 × 8.0 mm^3^, TI1 2000 ms, bolus length 1,000 ms, repetition time (TR)/ echo time (TE) 4,500/23.58 ms, flip angle 180°, bandwidth 4,340 Hz/Px, slice thickness 8 mm, number of slices 8. The total scan time of the ASL sequence was 4 min 33 s. Background suppression pulse was used to suppress T1 values of 230 ms. At each time point two consecutive ASL scans were performed.

#### 2.5.2 Blood oxygen level-dependent

A multi-echo gradient-recalled echo (mGRE) sequence with 12 equally spaced echo times was used to obtain BOLD images. Consensus-based technical recommendations for clinical translation of renal BOLD MRI were followed [19]. The acquisition parameters of the BOLD sequence included the following: TR 70 ms, TEs 4.20–50.40 ms (echo time intervals 4.20 ms), matrix 96 × 96, FOV 180×180, number of slices 4, slice thickness 4 mm, coronal oblique plane, flip angle 30°, voxel size 1.9×1.9×4.0 mm, bandwidth 300 Hz/Px, fat saturation was applied. The parallel imaging technique GRAPPA (generalized autocalibrating partially parallel acquisitions) was used with an acceleration factor of 2. With an acquisition time of 13 s, each kidney was scanned once. Each scan was completed during a 15 s breath-hold, which was induced by applying end-expiratory positive pressure with the breathing balloon.

#### 2.5.3 Dynamic contrast-enhanced

For DCE MRI acquisition the RAVE (Radial Volumetric Encoding) sequence was used with the following parameters: TR/TE of 4.80/1.37 ms, flip angle of 17°, 36 slices, slice thickness of 3 mm, matrix of 256×128 mm, FOV of 380×380 mm, voxel size of 1.5×1.5×3.0 mm, bandwidth of 780 Hz/Px. A dose of 0.2 mL/kg of Gadolinium contrast agent (Clariscan^®^, 0.5 mmoL/mL, GE Healthcare, Nydalen, Oslo) was injected as a bolus at 1 mL/s followed by a 12 mL saline flush at the same rate using an automated contrast injector (BAYER Medrad^®^ Spectris Solaris EP).

### 2.6 Post-processing

#### 2.6.1 Arterial spin labeling

Label, control, and M0 images, as well as Perfusion-Weighted (PW) and RBF maps were generated inline on the scanner, preceded by motion correction that was applied to the acquired MR datasets using an elastic registration algorithm (Applications Guide, Arterial Spin Labeling Sequence for Kidney Perfusion Assessment, Siemens Healthineers, Erlangen DE). Using MRIcroGL (RRID:SCR_024413), all ASL DICOM images were converted to NIfTI format (61). The mid-coronal slice of each kidney was identified, and a whole kidney region of interest (ROI) was manually drawn by a fourth-year PhD student (A.H.) on the PW images with 3D Slicer (RRID:SCR_005619). RBF was determined by ASL-MRI using the average of two consecutive scans and is expressed in mL/100 g/min.

#### 2.6.2 Blood oxygen level-dependent

Four-dimensional images were produced by the scanner, with the first three dimensions representing the acquired volume and the fourth dimension representing the voxel value at different echo times. A voxel-by-voxel calculation was performed to generate estimated R2* maps following Zhang et al. (62). On a mid-coronal slice of the T2* weighted image, which served as an anatomic template, a mask covering the renal parenchyma of the whole kidney was manually defined by a fourth-year PhD student (A.H.) using 3D Slicer (RRID:SCR_005619).

#### 2.6.3 Regional image analysis

**Figure 3:**
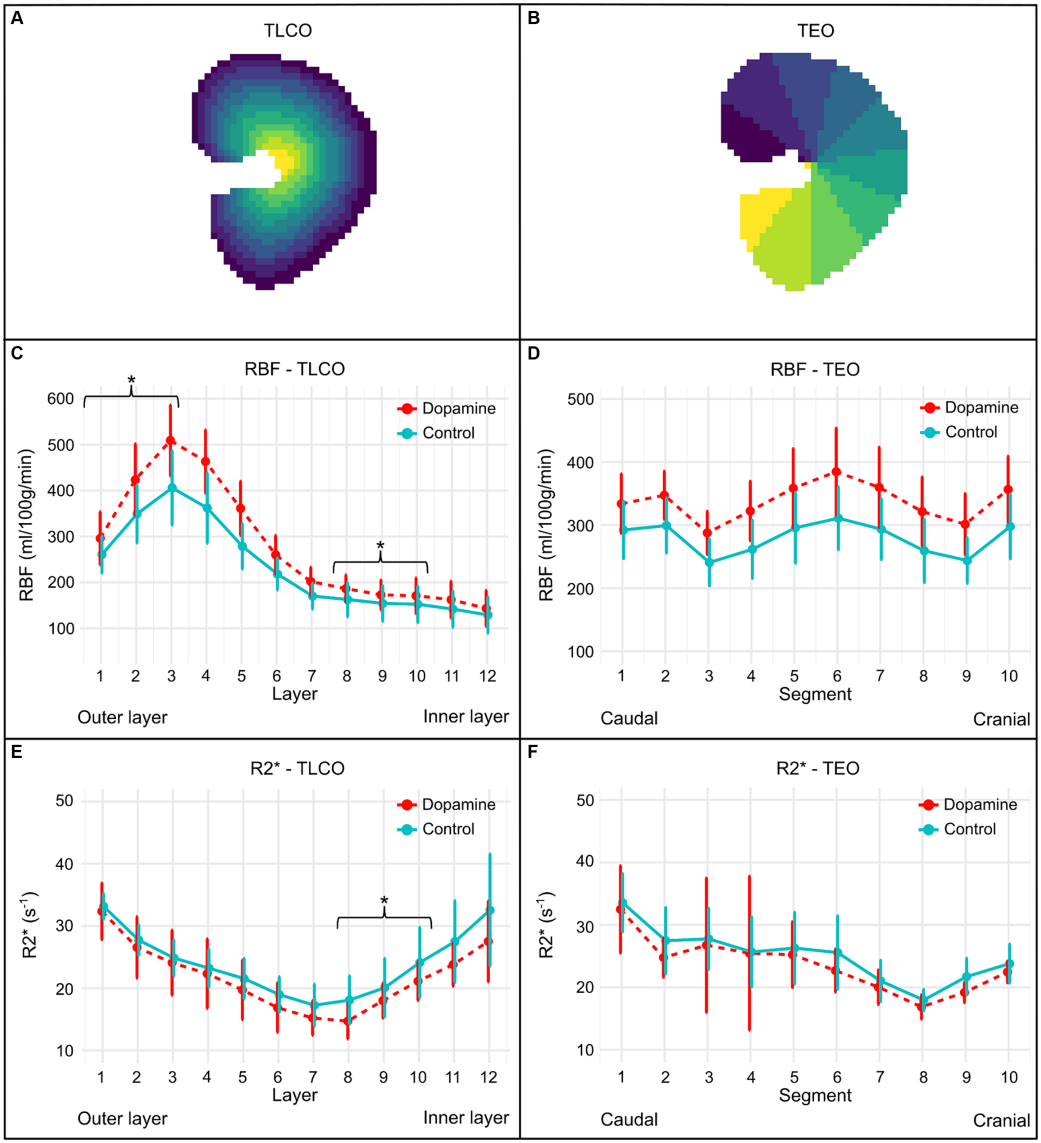
Illustration of the regional division of the kidney by the TLCO **(A)** and TEO method **(B)**, as well as the RBF **(C,D)** and R2* plots **(E,F)** of all subjects using the TLCO and TEO method. **(A)** The Twelve Layer Concentric Objects (TLCO) technique, which divides the renal parenchyma into 12 layers of uniform thickness. **(B)** The Ten Equiangular Objects (TEO) technique, which divides the kidney from the cranial to the caudal pole into ten equiangular segments. The RBF plots (mean ± SD) of all subjects during 0.9% NaCl infusion (control) and dopamine infusion using the TLCO **(C)** and TEO **(D)** method. The R2* plots (mean ± SD) of all subjects during 0.9% NaCl infusion (control) and dopamine infusion using the TLCO **(E)** and TEO **(F)** method. In the TLCO method, layer 1 refers to the outer layer and layer 12 to the inner layer. In the TEO method, the segment at the caudal pole is represented by 1 and the segment at the cranial pole by 10. ^*^p-value <0.05.

Analysis of ASL and BOLD images was conducted using the 12-layer concentric objects method (TLCO) and TEO method (Ten Equiangular Object) (45, 46, 63). With the TLCO-method, an automatic algorithm divides the renal parenchyma selected by the whole kidney ROI into 12 concentric layers of equal thickness (45, 46). The novel approach called the TEO method divides the kidney into ten equiangular segments from the cranial to the caudal pole, enabling evaluation along the renal cortex (63). Figures 3A,B illustrates the division of the kidney into twelve concentric layers using the TLCO method and in ten equiangular segments using the TEO method.

The TLCO-method involves calculating a center of mass C(x,y) for each mask (64). Each point P(x,y) within the mask is classified into 12 equal sections based on its distance from C. By implementing the TLCO method in Python [Python Programming Language (RRID:SCR_008394)], a scientific software development environment, the average perfusion and oxygenation values at different distances from the center were calculated. As a result, a label image with 12 concentric layers was obtained as well as the average RBF and R2* values for each layer. Values for the cortex, medulla, and whole kidney have been estimated based on the measurements of these 12 layers, using the following definitions: cortex (mean of the three outer layers), medulla (mean of the inner 8th to 10th layer) and whole kidney (mean of all 12 layers) (45). The TEO method, implemented in Python [Python Programming Language (RRID:SCR_008394)], defined 10 regions. A label image with 10 equiangular objects was obtained. The mean perfusion rates and oxygenation levels were calculated using TEO across the different kidney sections.

#### 2.6.4 Dynamic contrast-enhanced

All processing was carried out offline. Image data from DCE was transferred in DICOM format and reconstructed with Golden-angle Radial Sparse Parallel imaging (GRASP) (65). The reconstructed images were analyzed with PMI 0.4, written in IDL 6.4 (RRID:SCR_025084).

**Figure 4:**
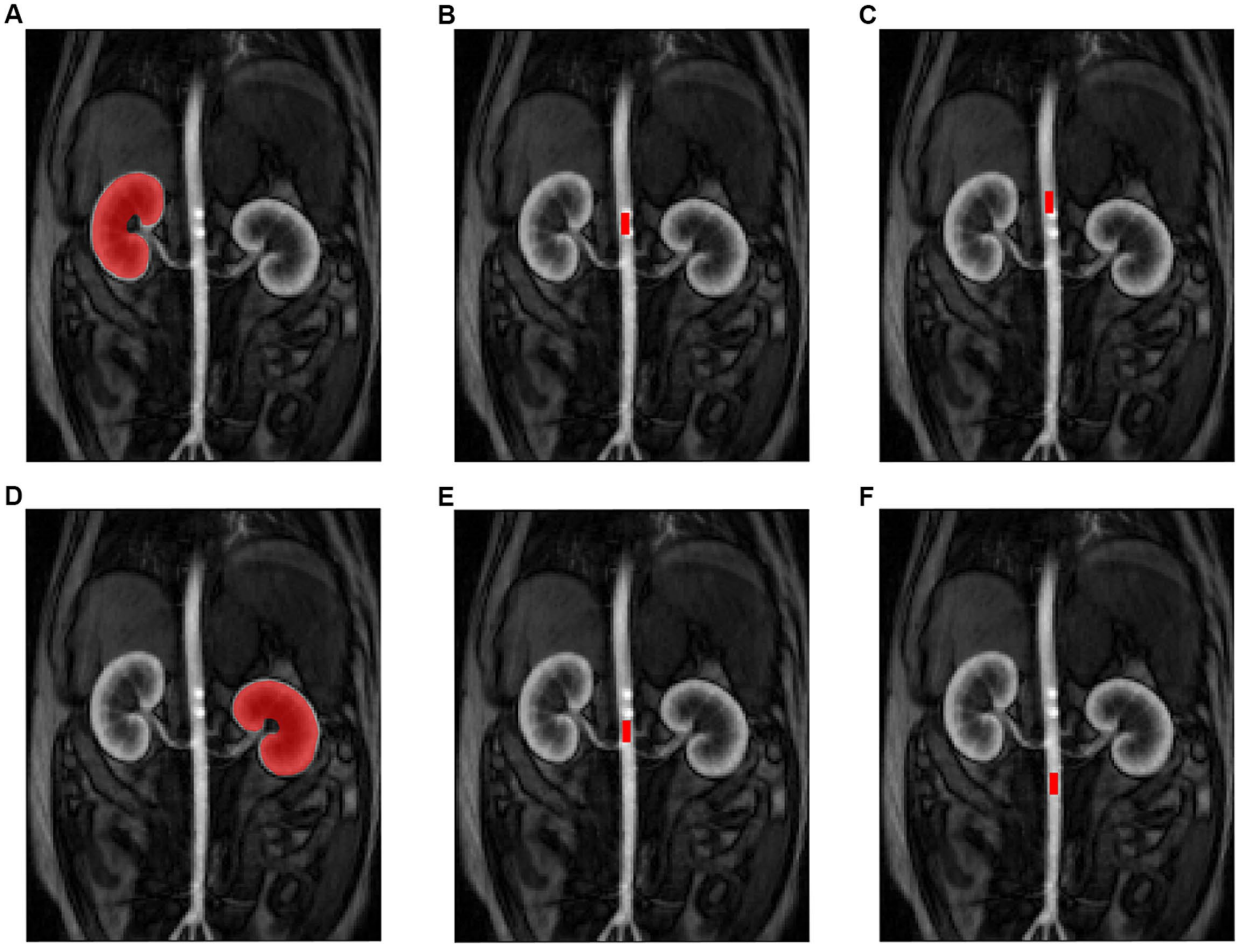
Illustration of the MRI region of interest (ROI) settings for the kidneys and arterial input function (AIF). Placement of the renal ROI on the right **(A)** and left **(D)** kidney. Placement of the AIF ROI: 12 mm cranial to the right renal artery branch **(B)**, 25 mm cranial to the right renal artery branch **(C)**, just caudal to the celiac and cranial mesenteric arteries **(E)** and 12 mm caudal to the right renal artery branch **(F)**.

The arterial input function (AIF) in this study describes the changes in contrast agent concentration over time in the abdominal aorta supplying blood to the kidneys. Four different placements for the ROI in the aorta to define the AIF were evaluated in this study (Figure 4). The AIF ROIs were rectangular and had a standardized size (10 × 4.4 mm). The first and second ROI were placed 12 and 25 mm cranial to the right renal artery branch, respectively. The third ROI was also located cranial to the right renal artery, but just caudal to the celiac and cranial mesenteric arteries to avoid this area of increased intensity. The fourth ROI was placed 12 mm caudal to the right renal artery branch. A renal ROI was drawn around the left and right kidney, respectively. The renal ROIs were defined on the middle coronal slice of the perfusion image of the respective kidney. For RBF determination following compartment models were evaluated: C2 filtration model (C2FM), C2 uptake model (C2UM), model-free (MF) and maximum slope (MS).

#### 2.6.5 Kidney volume

For kidney volume determination, manual segmentation on each T1 VIBE (Volume Interpolated Breath-hold Examination) image slice was performed using 3D Slicer (RRID:SCR_005619). The software then generated a renal volume automatically. The sequence parameters of the T1 VIBE ISO sequence were: FOV 380 mm, voxel size: 1.0×1.0×1.0 mm, slice thickness: 1.0 mm, TR/TE: 3.64/ 1.45 ms, flip angle: 9.0°, number of slices: 144, bandwidth 450 Hz/Px.

### 2.7 Statistics

Statistical analyses were done in R version 4.3.2 (R Project for Statistical Computing (RRID:SCR_001905)). The analysis was done stepwise. First, correlations between techniques were calculated in the native phase, i.e., when the dogs did not receive treatment, using the spearman correlation coefficient. Based on the guidelines from Schober et al. (66), correlation was considered negligible if *r* was less than 0.10, weak if *r* was between 0.10 and 0.39, moderate if *r* was between 0.40 and 0.69, strong if *r* was between 0.70 and 0.89 and very strong if *r* was greater than 0.90. Next, the effect of receiving dopamine on various outcome parameters (ASL TLCO, ASL TEO, BOLD TLCO, BOLD TEO, DCE, CEUS, respectively) was evaluated using a linear mixed model with treatment as fixed effect and the appropriate random effect. For CEUS, the random effect was dog; for DCE, the random effect was dog and side nested within dog; for BOLD and ASL, the random effect was dog, side nested within dog and segment nested within side. In every model, significance was assessed with a likelihood ratio test. Finally, the association between kidney volume and treatment was evaluated with a linear mixed model with treatment as fixed effect and as random effects dog, and side nested within dog. Significance was set at *α* ≤ 0.05 and a Bonferroni-correction for multiple testing was applied when needed. Corrected *p*-values are reported.

## 3 Results

### 3.1 Pharmacokinetic model for dynamic contrast-enhanced-MRI

The uptake model and the model-free model were the only DCE-MRI PK models whose RBF measurements demonstrated a significant correlation with RBF measurements of ASL-MRI at baseline. With regard to the model-free model, this applied to analyses where the AIF ROI was located 25 mm cranially, 12 mm cranially, or caudally to the right renal artery. For the uptake model, this specifically concerned the analysis in which the AIF ROI was positioned 25 mm cranially to the right renal artery. For the different combinations of PK model and AIF ROI placement in DCE analysis, Table 1 shows the significance of the correlation between the measured RBF and the RBF measured with ASL-MRI.

**Table 1:** Overview of the significance of the correlations between RBF measured with ASL-MRI and RBF measured with DCE-MRI for different combinations of PK model and AIF ROI placement.

**ROI placement**	**Pharmacokinetic model**			
	C2UM	C2FM	MF	MS
Cr 25 mm	0.01^*^	1.01	0.02^*^	0.16
Cr 12 mm	0.85	3.19	0.05^*^	0.52
Ca	0.07	0.61	0.01^*^	0.17
AD	0.99	2.16	0.68	1.54

PK, Pharmacokinetic model; AIF, arterial input function; ROI, region of interest; C2FM, the two compartment filtration model; C2UM, the two compartment uptake model; MF, the model-free deconvolution model; MS, maximum slope model; Cr 25 mm, 25 mm cranial to the right renal artery branch; Cr 12 mm, 12 mm cranial to the right renal artery branch; Ca,12 mm caudal to the right renal artery branch; AD, cranial to the right renal artery, but just caudal to the celiac and cranial mesenteric arteries. ^*^p-value’s < 0.05.

### 3.2 Correlation between imaging methods at baseline

Between ASL- and BOLD-MRI measurements a significant negative correlation was found at the level of the cortex (*r* = −0.42, *p* < 0.01) and the medulla (*r* = −0.46, *p* = 0.001). So there is an inverse relation between renal perfusion and R2*, R2* decreases with an increasing RBF. This suggests that increased renal perfusion is accompanied by increased renal oxygenation.

Table 2 summarizes the correlations of RBF and R2* with the CEUS parameters at different levels of the kidney. A significant positive correlation was observed for blood volume parameters WiR and PI with RBF, both at the level of the cortex and medulla. The RBF likewise revealed a significant positive correlation with blood volume parameter AUC and blood velocity parameter FT, however, only at the cortex level. On the contrary, a negative correlation was found between blood velocity parameter RT and RBF at the medulla. Based on these findings, elevated renal blood volume and increased rate of blood inflow are related to higher renal blood flow.

**Table 2:** Correlation coefficients of RBF and R2* with CEUS parameters at the cortex and the medulla.

	Correlation RBF – CEUS parameters		Correlation R2* – CEUS parameters	
Parameter by location	*r*	*p*-value	*r*	*p*-value
Renal cortex				
TTP	−0.1	1	0.11	1
FT	0.47	0.005^*^	−0.03	1
RT	−0.17	1	0	1
PI	0.41	0.02^*^	−0.09	1
AUC	0.43	0.02^*^	−0.08	1
WiR	0.38	0.05^*^	−0.08	1
Renal medulla				
TTP	−0.19	1	0.22	0.84
FT	0.09	1	0.03	1
RT	−0.41	0.03^*^	0.29	0.26
PI	0.49	< 0.01^*^	−0.45	< 0.01^*^
AUC	0.16	1	−0.17	1
WiR	0.68	< 0.001^*^	−0.42	0.02^*^

r, correlation coefficient; TTP, time to peak [s]; FT, fall time [s]; RT, rise time [s]; PI, peak intensity [a.u]; AUC, area under the curve [a.u * s]; WiR, wash-in rate [a.u/s]; a.u, arbitrary units; s, seconds. ^*^p-value’s < 0.05.

For R2*, significant negative correlations were found with PI and WiR at the medulla. At the cortex, however, no significant correlations were observed between R2* and any of the CEUS parameters. These results suggest that increased medullary renal blood volume is related to a higher oxygenation.

### 3.3 Pharmacological challenge with dopamine

The mean ± SD RBF values during 0.9% NaCl infusion (control) and dopamine infusion of each layer using the TLCO method and of each segment obtained using the TEO method is illustrated in Figures 3C,D. Dopamine infusion induced a significant increase in RBF at the level of the cortex (*p* < 0.001; ∆RBF = +71.15 mL/100 g/min), medulla (*p* < 0.001; ∆RBF = +20.83 mL/100 g/min) and whole kidney (*p* < 0.001; ∆RBF = +47.58 mL/100 g/min), as observed with the TLCO method. A significant increase in RBF was also observed with the TEO method for the whole kidney during dopamine infusion compared to 0.9% NaCl infusion (*p* < 0.001; ∆RBF = +54.31 mL/100 g/min).

Figures 3E,F illustrates the mean ± SD R2* values during 0.9% NaCl infusion (control) and dopamine infusion of each layer using the TLCO method and of each segment obtained using the TEO method. With the TLCO method, a lower R2* was observed at the cortex, medulla and whole kidney during dopamine infusion compared to 0.9% NaCl infusion. However, a significant decrease in R2* was only observed at the medulla (*p* < 0.01; ∆R2* = −2.83 s^−1^) and the whole kidney (*p* < 0.001; ∆R2* = −2.19 s^−1^). The difference in R2* at the cortex was −0.72 s^−1^ (*p* = 0.50). With the TEO method, the whole kidney had a lower R2* during dopamine infusion compared to 0.9% NaCl infusion, but this difference was not significant (*p* = 0.08; ∆R2* = 1.36 s^−1^).

Table 3 summarizes the mean RBF (mean of 2 runs) and R2* during 0.9% NaCl infusion (control) and dopamine infusion of the renal cortex, medulla, and the whole kidney in both kidneys using the TLCO method.

**Table 3:** Regional mean and standard deviation of RBF and R2* during 0.9% NaCl infusion (control) and dopamine infusion derived with the TLCO method.

Parameter by location	Dopamine	Control	*p*-value
Renal cortex			
RBF	409.87 ± 113.25	338.72 ± 87.43	<0.001*
R2*	27.86 ± 6.01	28.58 ± 4.26	0.500
Renal medulla			
RBF	177.46 ± 34.48	156.63 ± 39.13	<0.001*
R2*	17.94 ± 3.93	20.77 ± 5.43	<0.010*
Whole kidney			
RBF	279.79 ± 134.63	232.21 ± 105.69	<0.001*
R2*	21.90 ± 6.73	24.09 ± 6.85	<0.001*

RBF, renal blood flow [ml/100 g/min]; R2*, apparent relaxation rate [s^−1^]; *p-value’s < 0.05.

**Figure 5:**
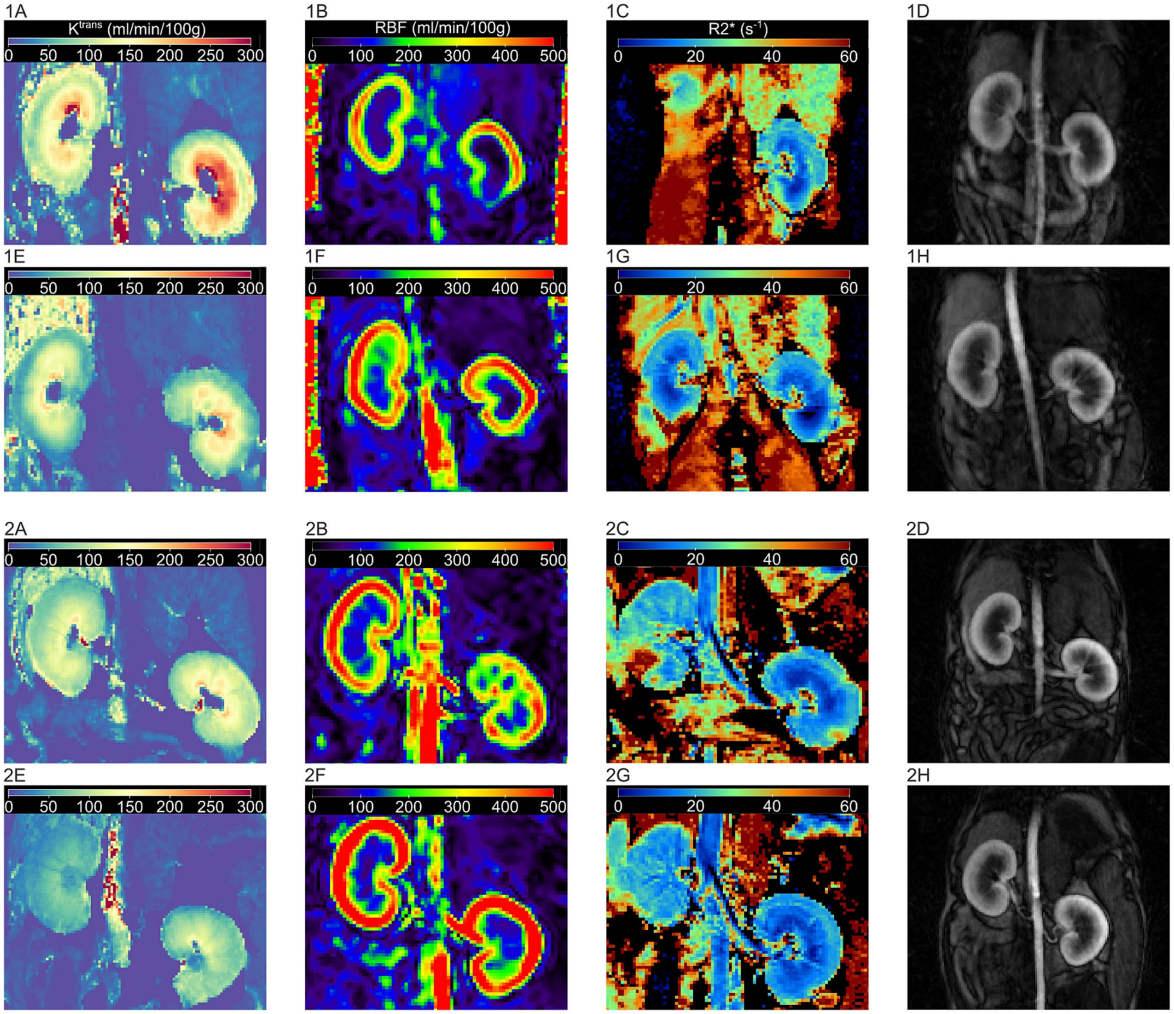
Illustration of DCE-MRI images and MRI maps for K^trans^, RBF and R2* during control and dopamine treatment for two subjects. 1: subject one, 2: subject two. MRI images during control treatment: **(A)** K^trans^ map, **(B)** RBF-map, **(C)** R2* map, and **(D)** DCE image. MRI images during dopamine treatment: **(E)** K^trans^ map, **(F)** RBF-map, **(G)** R2* map, and **(H)** DCE image. Ktrans: volume transfer constant, RBF: renal blood flow, R2*: apparent relaxation rate, DCE: dynamic contrast enhanced.

Of the DCE-MRI analysis methods that showed a significant correlation with ASL-MRI, only the model-free model with the ROI caudal to the right renal artery could detect a significant difference in RBF induced by dopamine. This DCE-MRI analysis method showed a significantly higher RBF (∆RBF = 100.65, *p* < 0.05) during dopamine infusion than 0.9% NaCl infusion. In Figure 5 the DCE-MRI images, as well as the K^trans^, RBF and R2* maps are presented of two subjects during each treatment.

**Figure 6:**
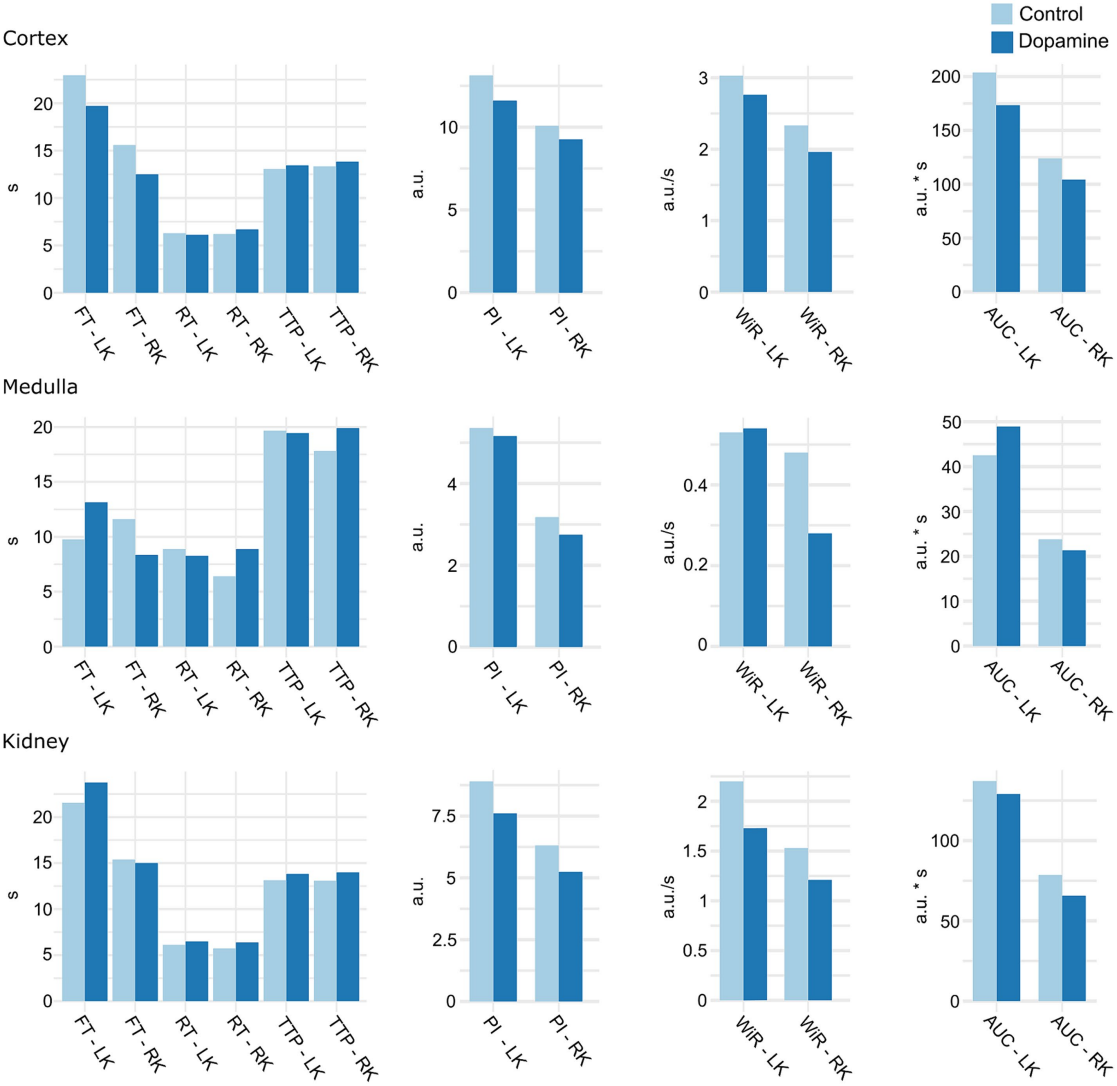
Mean of CEUS renal perfusion parameters during 0.9% NaCl infusion (control) and dopamine infusion. LK, left kidney; RK, right kidney; TTP, time to peak; FT, fall time; RT, rise time; PI, peak intensity; AUC, area under the curve; WiR, wash-in rate; a.u, arbitrary units; s, seconds. There were no significant differences.

Quantitative CEUS showed no significant differences in renal perfusion parameters between dogs receiving a 0.9% NaCl and dopamine infusion. Figure 6 shows an overview of the renal perfusion parameters during 0.9% NaCl infusion and dopamine infusion. In addition, no significant effect of dopamine was observed on kidney volume (*p* = 0.60).

## 4 Discussion

This study was conducted to evaluate the potential role of the emerging imaging technique fMRI in assessing renal perfusion and oxygenation in dogs. Among the DCE-MRI analyses methods, the model-free model with caudal placement of the AIF ROI in the aorta showed most potential in dogs. Additionally, R2* negatively correlated with ASL measured RBF at the cortex and medulla, as well as with medullary WiR and PI. ASL measured RBF, on its turn, showed a positive correlation with cortical WiR, PI, AUC and FT, and with medullary WiR and PI, but a negative correlation with medullary RT. During a pharmacological challenge with dopamine, BOLD-MRI observed a significant decrease in R2* (higher oxygenation) at the medulla and entire kidney, while ASL-MRI demonstrated a significant increase in renal perfusion at the cortex, medulla and the entire kidney.

In renal DCE-MRI, a bolus of gadolinium-based contrast agent (CA) is injected intravenously and its progression through the kidney is tracked by acquiring a time series of T1-weighted images (21, 22). Applying a pharmacokinetic (PK) model to the dynamically acquired tissue concentration curve allows quantification of physiological parameters like RBF and GFR (21, 22). Several PK models have been described for determining renal perfusion and filtration based on DCE-MRI data, but there is no consensus about which model is most suitable (21, 22). Among the models to measure renal perfusion are the two-compartment filtration model (C2FM), the two-compartment uptake model (C2UM), the model-free deconvolution model (MF) and maximum slope model (MS) (22). All of these PK models require measurements of the arterial input function (AIF), which describes the changes in CA concentration over time in a major feeding artery of the kidney, usually the aorta (21, 22). The AIF is defined by the MRI signal obtained from a selected region in the aorta (22). The size and placement of this region in the aorta, however, affect renal perfusion values significantly (67, 68). Nevertheless, there is also no agreed standard for drawing the ROI in the aorta for AIF determination (67, 68). In the present study, the uptake model and the model-free model yielded results that were significantly correlated with RBF measured by ASL-MRI. As for the model-free model, this specifically pertains to analyses where the AIF ROI was positioned 25 mm cranial, 12 mm cranial, or caudal to the right renal artery. For the uptake model, this specifically concerned the analysis in which the AIF ROI was positioned 25 mm cranial to the right renal artery.

There was a significant correlation between several CEUS parameters and RBF determined by ASL-MRI at baseline. At the cortex, WiR, PI, AUC and FT were positively correlated with RBF. At the medulla, WiR and PI correlated positively with RBF, while RT showed a negative correlation. This was in line with the expectations as both RBF and CEUS parameters reflect renal perfusion, although CEUS provides a semi-quantitative assessment (16). With CEUS, a graph of contrast intensity versus time is generated following a bolus injection of contrast agent, from which parameters that semi-quantitatively describe blood perfusion are derived (16). The CEUS parameters extracted from the TIC curve can be divided into two categories: intensity-related parameters that reflect blood volume and time-related parameters that reflect blood velocity (16). The correlations in the current study therefore indicate that elevated renal blood volume and a faster rate of blood inflow are related to increased renal blood flow (16).

A moderate negative correlation between R2* and RBF determined by ASL-MRI was observed at baseline for the cortex and the medulla. As R2* reflects the absolute amount of deoxy-Hb per tissue volume, the negative correlation between R2* and RBF indicates: the lower the perfusion blood flow, the higher the deoxyhemoglobin content and therefore the lower the renal oxygenation (35). A reduction in renal blood flow leads to a decrease in oxygen delivery, which in turn lowers the oxyhemoglobin to deoxyhemoglobin ratio and results in a higher R2*. In human renal transplant patients, Peng et al. (69) also found a negative correlation between ASL and BOLD parameters. There was also a negative correlation between R2* and the CEUS parameters PI and WiR, but only at the level of the medulla. This further indicates that there is a relationship between decreased medullary perfusion and lower oxygenation.

Dopamine is a catecholamine that functions as a neurotransmitter and a hormone. Dopamine is a regulator of the circulatory, renal, and neurohumoral system, showing a dose-dependent effect. At low doses (≤3 μg/kg/min), dopamine increases renal blood flow, GFR, and sodium excretion through dilatation of renal vessels and inhibition of tubular ion reabsorption (70). At medium doses (3 to 7 μg/kg/min), dopamine additionally induces systemic hemodynamic changes such as increases in cardiac output, heart rate and reduction in peripheral resistance (70). At high doses (> 10 μg/kg/min), dopamine exerts a reverse effect through stimulation of α-adrenergic receptors. These high doses stimulate sodium and water reabsorption and induce vasoconstriction, resulting in decreased sodium excretion, urinary output, renal blood flow and GFR (70). Medium and high dopamine dose infusions can provoke arrhythmia, which requires careful subject monitoring (71). In this study, the dose of dopamine was determined based on previous studies in dogs investigating the effects of dopamine on kidney functions and hemodynamics (71–73). Dopamine at a low dose was chosen since higher doses do not induce a greater change in renal perfusion, but do affect systemic hemodynamics and cause arrhythmias, posing a greater anesthetic risk (71–73). In veterinary medicine, dopamine is used in the prevention and treatment of kidney and heart disease (72). Recently, however, researchers have questioned dopamine’s effectiveness in treating these conditions (70).

In this study, ASL-MRI was able to measure a significant increase in renal blood flow following dopamine infusion compared to control in the whole kidney, cortex and medulla. The magnitude of that increase was greater in the cortex than in the medulla. On average, renal perfusion during dopamine infusion increased by 20.1, 19.6 and 13.3%, at the entire kidney, cortex and medulla, respectively. The extent of dopamine’s effect on renal perfusion is subject to variation in the literature. In agreement with our results, a previous study investigating dopamine arrhythmogenicity using an electromagnetic flowmeter reported an increase in RBF of approximately 20% in dogs under anesthesia following a dose of 3 μg/kg/min (71). However, at the same dose, another canine study using colored microspheres observed an increase in RBF of 67.63 ± 23.08% (72). In healthy human volunteers, dopamine infusion at 3 g/kg/min significantly increased the effective renal plasma flow (ERPF) by 25–50% as measured with urine or plasma clearance of radioiodine-labelled-hippuran (74, 75).

Using BOLD-MRI, a decrease in R2* could be detected during dopamine infusion compared to 0.9% NaCl infusion, suggesting an increase in renal oxygenation. The difference in R2* reached statistical significance in the medulla and whole kidney, but not in the cortex. On average, dopamine infusion reduced the medullary R2* by 20% and the total renal R2* by 9.1%. The absence of a significant decrease in R2* for the cortex, in contrast to the medulla, may be due to the lower sensitivity of BOLD-MRI to detect changes in cortical oxygenation (47). This is because of the different relative positions of medullary and cortical oxygenation on the oxygen dissociation curve (47). Consequently, a greater change in oxygenation is required to produce a similar change in R2* for the cortex as for the medulla (47). Our results are in line with those by Redfors et al. (75) who examined the impact of low-dose dopamine on renal hemodynamics and oxygenation in humans, post-cardiac surgery. Their research has shown that dopamine increases renal oxygenation by 28–34% together with a 45–55% increase in RBF (75, 76).

The assumption that dopamine always increases renal oxygen consumption is contradicted by the findings of the current study and the study conducted by Redfors et al. (75) and Ricksten et al. (76). Dopamine administration was thought to increase renal oxygen consumption because it would increase the amount of Na that is supplied to the tubular cells for active transport-mediated reabsorption (75, 76). Dopamine was suggested to increase Na load in the tubular filtrate in two different ways. Firstly, by inhibiting Na reabsorption in the proximal tubule, more Na would flow into the distal tubule possibly increasing regional oxygen consumption (76). Secondly, a rise in renal blood flow due to dopamine-induced vasodilation was expected to coincide with a proportional increase in GFR and sodium filtration (75). However, the pronounced increase in renal blood flow found in the study by Redfors et al. (75) was not accompanied by an increase in GFR. Renal oxygen consumption remained unaffected as a result of dopamine’s lack of influence on GFR, leading to improvements in renal oxygenation (75). Most likely, balanced vasodilation of pre- and post-glomerular arterioles is responsible for the dopamine-induced increase in renal blood flow without significant effects on GFR (75, 76). *In vitro* experiments using isolated rabbit renal arterioles have shown that dopamine mainly acts on dopamine-1 receptors, inducing equal dilatation of afferent and efferent arterioles (77). This probably explains the findings of our study as well.

In the present study, renal perfusion parameters of CEUS did not show a significant difference between 0.9% NaCL infusion and dopamine infusion. In contrast, CEUS has demonstrated the ability to detect drug-induced renal perfusion changes in previous studies. Several human studies showed that CEUS can identify changes in renal perfusion caused by pharmaceuticals (78, 79). In dogs, contrast-enhanced power Doppler imaging detected significant changes in perfusion parameters induced by norepinephrine (80). It must be highlighted, however, that the magnitude of the significant changes induced was substantial, ranging from 47 to 92% in these human studies (78, 79) and 62 to 94% in the canine study (80). In dogs, the anticipated rise in RBF due to dopamine varies from 20 to 50% (71, 72). Possibly the dose of dopamine used did not affect renal perfusion sufficiently for a significant difference to be observed with CEUS. In fact, CEUS is known for its significant variability in the quantification of tissue perfusion, which is primarily due to patient-related factors, scanner settings, and contrast agent-related factors (81). The high inherent variability of CEUS leads to diagnostic uncertainty (81).

Among the DCE analysis methods significantly correlated with RBF measured by ASL-MRI, only the model-free model with the ROI caudal to the right renal artery could detect a significant difference in RBF with dopamine infusion compared to 0.9% NaCl infusion. These results may indicate that the model-free model with placement of the AIF ROI caudal to the right renal artery has great potential to assess renal perfusion in the dogs. However, additional research is required to determine the most suitable DCE analysis method for dogs. In future studies, it would be beneficial to use a golden reference method to calculate the true RBF.

There were some limitations to the present study. First, reference standards for quantifying renal blood flow, renal oxygenation, and GFR were not included in this study. The current gold standard methods for measuring renal blood flow and tissue oxygenation are invasive, requiring surgical intervention or animal euthanasia. These techniques were not used for animal welfare reasons. It should be noted, however, that the imaging techniques used in this study have previously been validated in animal studies against gold standard techniques. ASL-MRI has been compared with microspheres (38) and ultrasound flowmeter (37) in a porcine model, as well as with histology in rodent models (82) which all showed a good correlation. Comparisons of BOLD-MRI with oxygen-sensitive microelectrodes in multiple pig models have demonstrated that regional oxygen content of the kidneys can be reliably estimated by BOLD-MRI (48, 49). For DCE-MRI, a good correlation has been established between measurements of renal perfusion and those determined with colored microspheres in swine (83) and transit-time flow probes in dogs and rabbits (32, 84). A second limitation might be that only one mid-coronal slice was used to determine ASL-, BOLD-, and DCE-MRI biomarkers. Since the medullary volume fraction of the kidney is higher in a mid-coronal slice than in peripheral slices, this approach might not be an exact representation of the whole kidney (85). However, a study by Winter et al. (86) found that DCE-MRI perfusion parameters from a single slice yielded similar values to those obtained from whole kidneys in humans. Furthermore, in the current study, perfusion and oxygenation were also determined for the cortex and medulla separately with ALS- and BOLD-MRI. Another limitation is that the dopamine infusion and control infusion were performed two to 3 weeks apart. This is because DCE-MRI requires the use of a contrast agent; hence, repeating the MRI scans on the same day would have resulted in erroneous readings due to the residual contrast agent still present in the body. Moreover, the fMRI scan and CEUS examination were performed on two consecutive days since they were performed at different locations. As a result of the time interval between the different examinations, changes in renal blood flow and oxygenation may have been caused by spontaneous fluctuations or time-dependent effects. However, several systemic hemodynamic parameters, including blood pressure, were regularly monitored during the examinations to eliminate any possibility that these factors might have been responsible for the changes observed in renal blood flow or oxygenation.

## 5 Conclusion

In conclusion, ASL- and BOLD-MRI can measure pharmacologically induced changes in renal blood flow and renal oxygenation in dogs. In contrast, no significant changes in renal perfusion parameters could be observed with CEUS. This might indicate that fMRI modalities allow for the detection of changes that cannot be observed with CEUS. Our results further indicate a correlation between renal perfusion and oxygenation. Regarding DCE-MRI, the model-free model with placement of the AIF ROI caudal to the right renal artery appears to be a promising analysis method in dogs. The use of multiparametric MRI can provide insight into (patho) physiological processes in the kidney and evaluate treatment outcomes. However, further research is needed to confirm the potential of ASL- and BOLD-MRI in dogs and to clarify which analysis method is most suitable for DCE-MRI in dogs.

## Data availability statement

The original contributions presented in the study are included in the article/Supplementary material, further inquiries can be directed to the corresponding author.

## Ethics statement

The animal study was approved by the Animal Ethics Committee from the Faculty of Veterinary Medicine and the Faculty of Bioscience Engineering of Ghent University, Belgium (Approval number: EC2021-085). The study was conducted in accordance with the local legislation and institutional requirements.

## Author contributions

AH: Writing – review & editing, Writing – original draft, Visualization, Project administration, Methodology, Investigation, Funding acquisition, Formal analysis, Data curation, Conceptualization. LS: Writing – review & editing, Visualization, Validation, Software, Resources, Methodology, Investigation, Formal analysis, Data curation, Conceptualization. SB: Writing – review & editing, Resources, Investigation, Data curation. BB: Conceptualization, Writing – review & editing, Validation, Methodology, Formal analysis. MH: Writing – review & editing, Resources, Conceptualization. EV: Writing – review & editing, Investigation. JG: Writing – review & editing, Investigation. ES: Writing – review & editing, Supervision, Methodology, Investigation, Funding acquisition, Conceptualization. PP: Software, Resources, Investigation, Writing – review & editing, Supervision, Methodology, Conceptualization. KV: Funding acquisition, Writing – review & editing, Supervision, Methodology, Conceptualization.

## Supplementary material

SUPPLEMENTARY DATA SHEET S1 Excel file with physiological parameters of the test subjects during MRI examination.

SUPPLEMENTARY DATA SHEET S2 Excel file with renal blood flow measurements with ASL-MRI using the TEO and TLCO method.

SUPPLEMENTARY DATA SHEET S3 Excel file with renal blood flow measurements with DCE-MRI.

SUPPLEMENTARY DATA SHEET S4 Excel file with oxygenation measurements with BOLD-MRI using the TEO and TLCO method.

SUPPLEMENTARY DATA SHEET S5 Excel file with physiological parameters of the test subjects during CEUS examination.

SUPPLEMENTARY DATA SHEET S6 Excel file with renal blood flow parameters measured with CEUS.
