# Supplementary material for: Assessment of pharmacologically induced changes in canine kidney function by multiparametric magnetic resonance imaging and contrast enhanced ultrasound
Source: Front Vet Sci. 2024 Jun 20;11:1406343. doi: 10.3389/fvets.2024.1406343 (PMC11223176; doi:10.3389/fvets.2024.1406343)
Supplement: Supplementary file 7 [file Table_1.docx]

Supplementary Material

Assessment of renal oxygenation and blood flow in dogs: A comparison of multiparametric MRI and contrast enhanced ultrasound

Amber Hillaert, Luis Carlos Sanmiguel Serpa, Stephanie Bogaert, Bart J. G. Broeckx, Myriam Hesta, Eva Vandermeulen, Jolien Germonpré, Emmelie Stock, Pim Pullens, Katrien Vanderperren^*^

*** Correspondence:** Katrien Vanderperren: Katrien.Vanderperren@ugent.be

# Supplementary Figures and Tables

Table S1. Mean and standard deviation of CEUS renal perfusion parameters during 0.9% NaCl infusion (control) and dopamine infusion. TTP, time to peak [s]; FT, fall time [s]; RT, rise time [s]; PI, peak intensity [a.u]; AUC, area under the curve [a.u]; WiR, wash-in rate [a.u]; a.u, arbitrary units; s, seconds.

|  | **Left kidney** | | | **Right kidney** | | |
| --- | --- | --- | --- | --- | --- | --- |
| **Parameter by location** | **Dopamine** | **Control** | **p** | **Dopamine** | **Control** | **p** |
| Renal cortex | | | | | | |
| TTP | 13.44 ± 1.27 | 13.07 ± 1.24 | 1 | 13.83 ± 1.16 | 13.33 ± 1.63 | 1 |
| FT | 19.72 ± 11.75 | 22.96 ± 11.88 | 1 | 12.51 ± 4.04 | 15.60 ± 8.45 | 1 |
| RT | 6.12 ± 0.79 | 6.29 ± 0.89 | 1 | 6.69 ± 1.20 | 6.20 ± 0.98 | 1 |
| PI | 11.61 ± 2.21 | 13.13 ± 1.59 | 1 | 9.28 ± 2.12 | 10.09 ± 2.47 | 1 |
| AUC | 173.47 ± 47 | 203.70 ± 85.66 | 1 | 104.36 ± 41.20 | 124.03 ± 75.02 | 1 |
| WiR | 2.76 ± 0.97 | 3.03 ± 1.07 | 1 | 1.96 ± 0.82 | 2.33 ± 0.93 | 1 |
| Renal medulla | | | | | | |
| TTP | 19.45 ± 1.64 | 19.67 ± 2.49 | 1 | 19.91 ± 3.27 | 17.82 ± 1.37 | 0.66 |
| FT | 13.15 ± 3.63 | 9.77 ± 3.13 | 0.34 | 8.35 ± 3.91 | 11.61 ± 5.16 | 1 |
| RT | 8.27 ± 1.51 | 8.88 ± 3.14 | 1 | 8.88 ± 2.06 | 6.40 ± 1.50 | 0.08 |
| PI | 5.16 ± 1.4 | 5.36 ± 1.60 | 1 | 2.75 ± 1.13 | 3.18 ± 1.32 | 1 |
| AUC | 48.93 ± 17.83 | 42.52 ± 22.36 | 1 | 21.36 ± 9.79 | 23.82 ± 15.90 | 1 |
| WiR | 0.54 ± 0.16 | 0.53 ± 0.24 | 1 | 0.28 ± 0.18 | 0.48 ± 0.28 | 0.34 |
| Whole kidney | | | | | | |
| TTP | 13.82 | 13.13 ± 1.08 | 1 | 13.99 ± 1.07 | 13.09 ± 1.43 | 1 |
| FT | 23.76 ± 9.92 | 21.56 ± 8.69 | 1 | 15.01 ± 5.79 | 15.38 ± 7.60 | 1 |
| RT | 6.48 ± 1.07 | 6.12 ± 0.76 | 1 | 6.39 ± 0.98 | 5.73 ± 0.69 | 0.79 |
| PI | 7.61 ± 2.44 | 8.90 ± 1.49 | 1 | 5.24 ± 2.22 | 6.31 ± 2.25 | 1 |
| AUC | 129.07 ± 70.46 | 137.12 ± 50.94 | 1 | 65.74 ± 45.98 | 78.59 ± 50.47 | 1 |
| WiR | 1.73 ± 0.72 | 2.20 ± 0.77 | 1 | 1.21 ± 0.73 | 1.53 ± 0.69 | 1 |
